# Electrochemical evaluation of stainless steel alloy as a ureteral stent in synthetic urine solution in-vitro with Candida urinary tract infection

**DOI:** 10.1186/s13065-025-01586-z

**Published:** 2025-07-26

**Authors:** Salma Mamdouh, Renad S. El-Kamel, Amany M. Fekry, Magda A. Ameer

**Affiliations:** Department of Chemistry, Cairo University, Faculty of Science, Giza, Egypt

**Keywords:** 316L stainless steel, Synthetic urine solution, Ureteral stents, *Candida albicans*, Stainless steel biofilm, Electrochemical corrosion

## Abstract

This study evaluates the electrochemical performance of 316 L stainless steel alloy as a potential ureteral stent material in a synthetic urine environment mimicking conditions of *Candida*-associated urinary tract infections. The electrochemical behavior was investigated using electrochemical impedance spectroscopy (EIS), cyclic voltammetry (CV), and potentiodynamic polarization techniques, both in the presence and absence of *Candida albicans*. Results demonstrated that the formation of a biofilm by *Candida albicans* significantly influences the corrosion behavior of the alloy, as evidenced by changes in electrochemical parameters. Surface morphology and microbial adhesion were further confirmed through scanning electron microscopy (SEM), revealing biofilm development on the alloy’s surface. These findings highlight the critical role of fungal colonization in the degradation and stability of metallic ureteral stents under infection conditions.

## Graphical Abstract

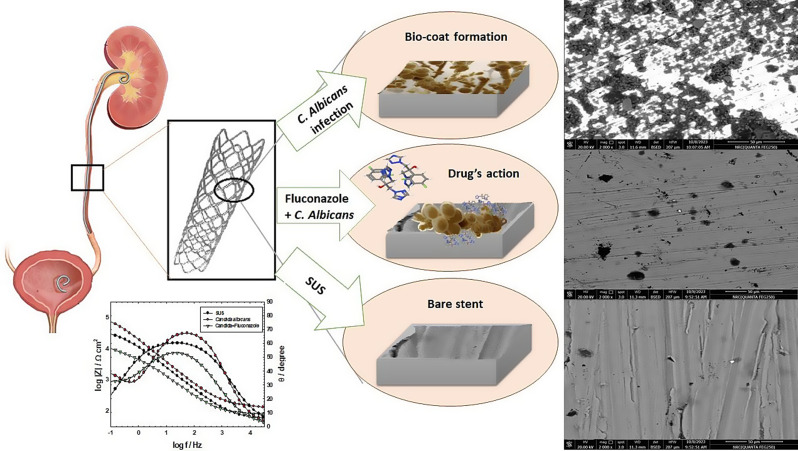

## Introduction

Managing ureteric strictures remains a significant clinical challenge. Various treatment approaches are available, including open surgical repair, minimally invasive procedures, and long-term stenting. To maintain urinary drainage in patients with ureteral strictures, Double-J stents and percutaneous nephrostomy tubes are commonly employed. However, these devices often require frequent replacement and can be associated with multiple complications [1].

Traditional polymeric stents have shown limitations, particularly in their ability to resist external compressive forces. Consequently, metal-based stents have been developed to offer improved mechanical durability. These are especially recommended in cases involving malignant extrinsic ureteral obstruction, where higher compression resistance is essential. Common materials used in metallic ureteral stents include stainless steel, nitinol, titanium-based superalloys, and cobalt-chromium alloys [2].

The Wallstent, constructed from a stainless steel wire mesh, was among the first metallic stents to demonstrate clinical efficacy in this context [3, 4]. Understanding the interaction between biomedical implants and their physiological environment is critical, especially with regard to the mechanical stability and biocompatibility of the materials used.

Among metallic biomaterials, 316 L stainless steel (SS316L) stands out for its high corrosion resistance. Its widespread application is attributed to its favorable mechanical properties, cost-effectiveness, biocompatibility, and ease of fabrication [5]. SS316L remains one of the most widely used alloys in the manufacture of reusable medical instruments and biomedical implants, including those for cardiovascular, orthopedic, dental, otolaryngologic, and craniofacial applications [6, 7]. While stainless steel implants are widely used, their long-term integrity in complex biological environments, particularly those challenged by persistent fungal infections, remains a significant concern, directly impacting patient outcomes and requiring further investigation. Some of the most prevalent bacterial infections are urinary tract infections (UTIs). Both Gram-positive and Gram-negative bacteria, in addition to specific fungi affect them mainly *Candida* species [8]. It is crucial to understand that *Candida albicans* (*C. albicans*) is the main cause of fungal UTI while investigating the conditions that make it possible for *Candida* species to cause UTI. 15–60% of people have *C. albicans*, which is a natural part of the body flora. The ability of *C. albicans* to colonize body locations close to or with access to the urinary tract is a factor in its potential as a pathogen of the urinary tract [9].

Previous studies [10–12] have primarily focused on bacterial biofilms, but the specific interactions and degradation mechanisms of metallic stents exposed to fungal UTIs have not been adequately investigated. To our knowledge, this is one of the first studies to systematically investigate the electrochemical performance of SS316L stents specifically under the influence of *C. albicans* biofilms and antifungal treatment in a simulated UTI model.

**Fig. 1:**
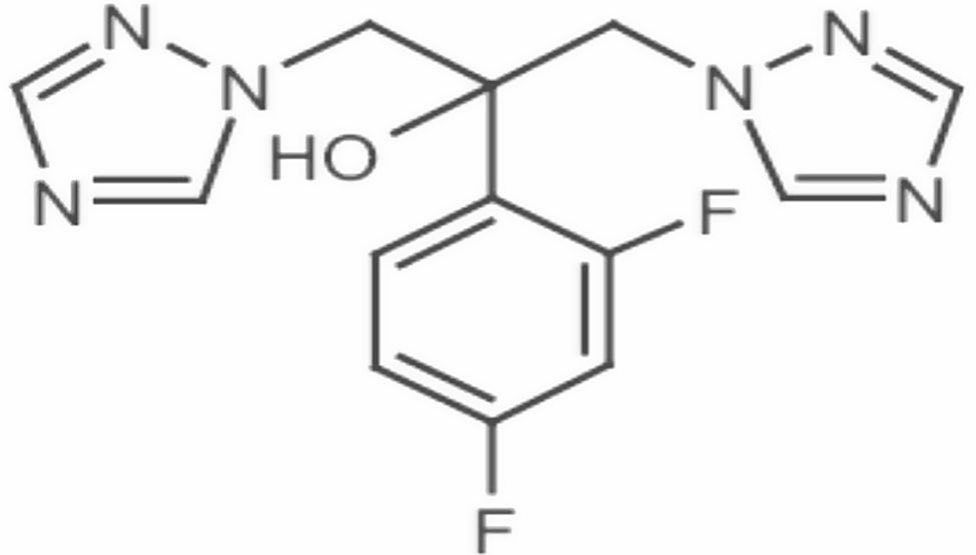
Molecular structure of Fluconazole

Fluconazole (Fig. 1), a triazole antifungal, is one of the most often recommended antifungal medications for *Candida* infections [13]. The azoles work by blocking the ergosterol biosynthesis pathway’s lanosterol demethylase (14α-demethylase) cytochrome P450 enzyme. This inhibition is hazardous because ergosterol is a crucial component of the fungal cell membranes and as a result, methylated sterols accumulate in the fungal cellular membrane and the cell growth of the fungus is ceased [14].

This study presents an innovative approach to evaluate the passivation behavior of SS316L alloy for potential use as a ureteral stent, with a particular focus on its in vitro biocompatibility with human cells. In parallel, a novel electrochemical sensor was developed by modifying a carbon paste electrode with electrodeposited gold nanoparticles (NAu-CPE). This sensor was specifically designed to detect the presence of the antifungal drug Fluconazole in simulated urinary systems. The structural and compositional characteristics of the materials were further confirmed through scanning electron microscopy (SEM) and energy-dispersive X-ray (EDAX) analysis.

## Experimental procedure

### Chemicals used

The chemicals used herein are sodium chloride, potassium chloride, sodium sulphate, potassium dihydrogen phosphate, ammonium chloride, calcium chloride and urea; they were all of analytical grade ≥ 98% (Sigma-Aldrich, USA). Fluconazole drug was bought from a local pharmacy (Flucoral capsules, 150 mg Fluconazole, SEDICO). *C. albicans* strains (from the National Collection of Industrial Microorganisms).

### Solution preparation

The synthetic urine solution (SUS) with pH 4.9 ± 0.1 was laboratory-prepared by dissolving the components in Table 1 in deionized water and was kept at -20 ^o^C until analysis [15].

**Table 1:** The chemical composition of SUS

Compound	Amount / g.L-1
NaCl	3.001
Na2SO4	2.017
KH2PO4	0.993
KCl	1.998
NH4Cl	1.0
CaCl2	0.755
Urea	25.225

### Alloy composition and preparation

The utilized alloy was in a rod shape with a circular exposed geometric area of 4 mm in diameter. Its components are as follows (in wt %): Cr = 16.71, Ni = 10.28, Mo = 2.07, C = 0.016, *N* = 0.067, Si = 0.48, S = 0.00006, *P* = 0.02, Cu = 0.12, Mn = 1.66 and balance Fe [16–22].

The electrode’s surface was pretreated by mechanical polishing with emery papers up to 1200 grit and then washed with ethanol and deionized water.

### Drug solution and bacteria preparation

The Fluconazole drug solution was prepared by emptying one capsule of the drug into 250 mL of SUS.

*C. albicans* strains was prepared by adding 1.0 mL of strains of activated solution into 25 mL of inactivated SUS, shaken well and incubated at a constant temperature of 37 °C, and the solution without microbial addition was used as the bare one. The sterile SS316L (working electrode) was incubated in the SUS (37 ⁰C) under anaerobic conditions for different times (1 h, 24 h, 120 h, 168 h, 336 h, and 408 h), at which the EIS curves measured, and then the polarization curves was measured at 408 h only for each modified electrode or the bare one.

### Instruments and cell used

The electrodes were tested by different techniques at different incubation times by means of an EC-Lab^®^ software SP-150 potentiostat, which was also employed to fit the experimental tests [23].

All experimental work was manifested in a 3-electrode cell [23] by applying the reference electrode (RE) to be a saturated calomel electrode (SCE), a platinum wire as the counter electrode (CE), and the SS316L as the working electrode.

EIS measurements were achieved in the frequency range of 0.1 Hz to 100 kHz using an AC amplitude excitation of 10 mV.

The pH was adjusted using an Adwa 1030 digital pH meter (Romania) [24].

Potentiodynamic polarization experiments were accompanied by 1 mV/s (scan rate) vs. SCE from − 0.6 V to -0.2 V.

### NAu-CPE preparation

**Scheme 1:**
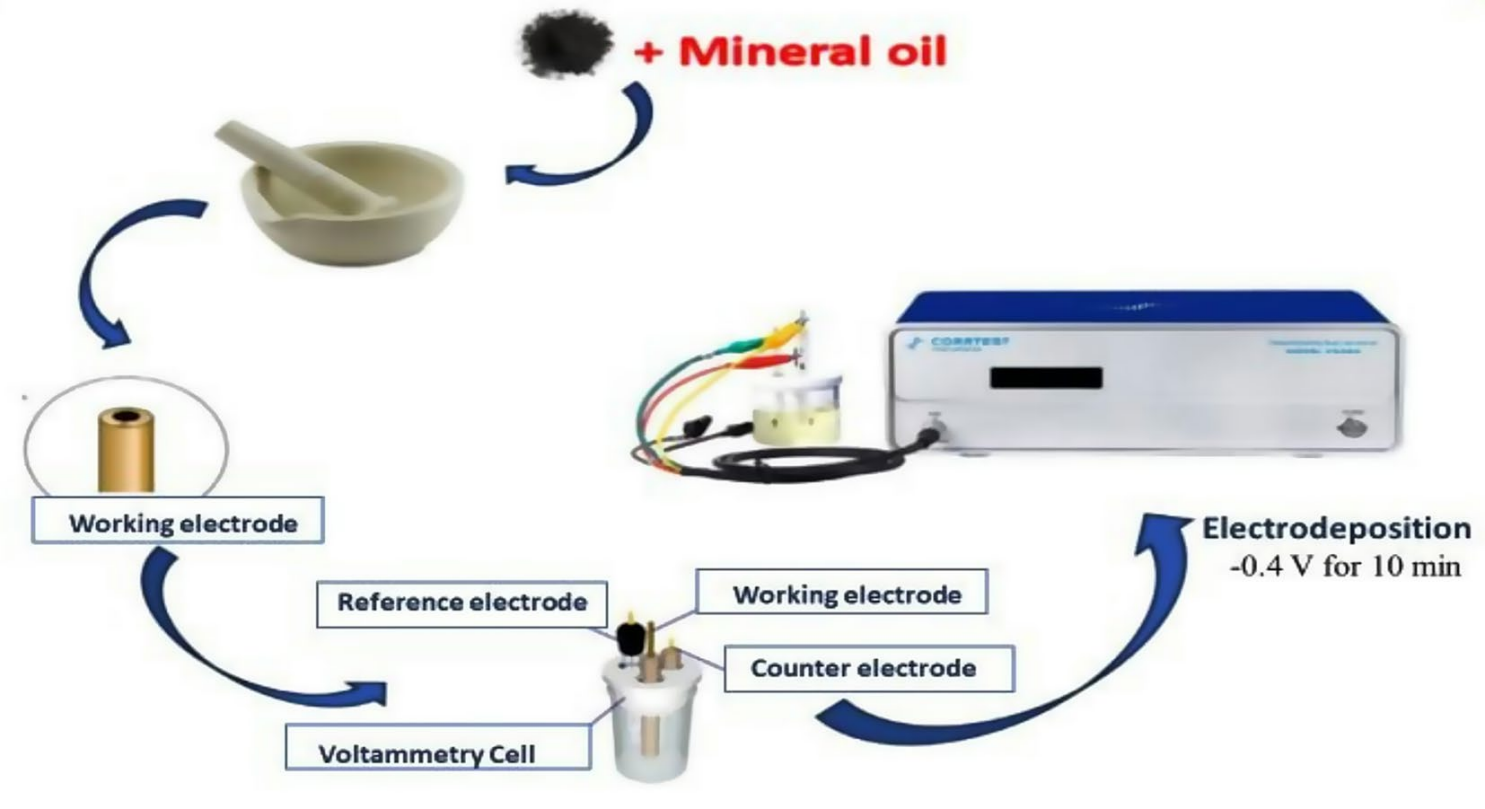
NAu electrodeposition on CPE

The carbon paste was prepared as previously mentioned in our work [25]. The NAu was electrodeposited on the electrode’s surface by dipping the electrode into gold solution (6 mM HAuCl4.4H2O in 0.1 M HNO3) and then applying − 0.4 V for 10 min [26]. This is best illustrated in Scheme 1.

### Surface characterization

Surface Characterization was identified by SEM Model Quanta 250 FEG (Field Emission Gun) fixed with EDAX element, with a speed up voltage of 30 kV, magnification 14x up to 1000000, and resolution for Gun 1 nm (FEI company, Netherlands) [23]. Raman spectroscopy was carried out using a LabRAM HR Evolution Raman microscope (Horiba), equipped with a laser source operating at an excitation wavelength of 532 nm. The exposure time for each measurement ranged from 3 to 5 s [27]. All tests were constructed three times at 37 °C and showed repeatable data.

## Results and discussion

*C. albicans* forms biofilms through surface adhesion and the secretion of extracellular polymeric substances, which can alter corrosion dynamics by creating protective or corrosive barriers. In this study, SS316L stainless steel samples were immersed in a synthetic urine at 37 °C for up to 408 h to simulate microbial colonization. To assess the impact of antifungal treatment, Fluconazole was introduced, and its influence on both biofilm stability and the corrosion behavior of the alloy was evaluated.

### Morphological characterization of SS316L electrode

**Fig. 2:**
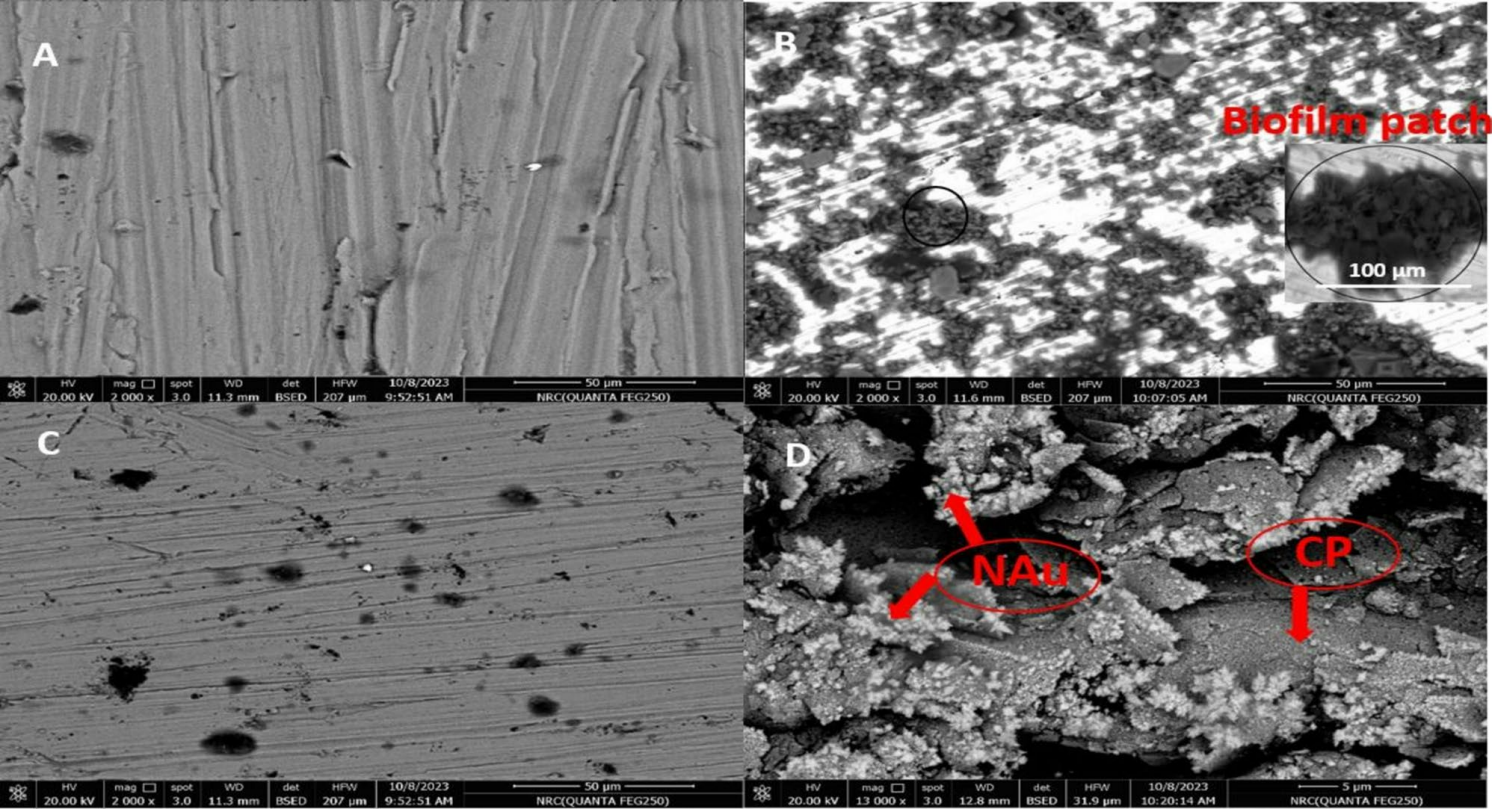
SEM images for the surface of the SS316L alloy in different media (**A**-**C**). SUS only (**A**), SUS containing *C. albicans* (**B**), SUS with *C. albicans* and Fluconazole (**C**). (**D**) SEM image for the surface of NAu-CPE

**Fig. 3:**
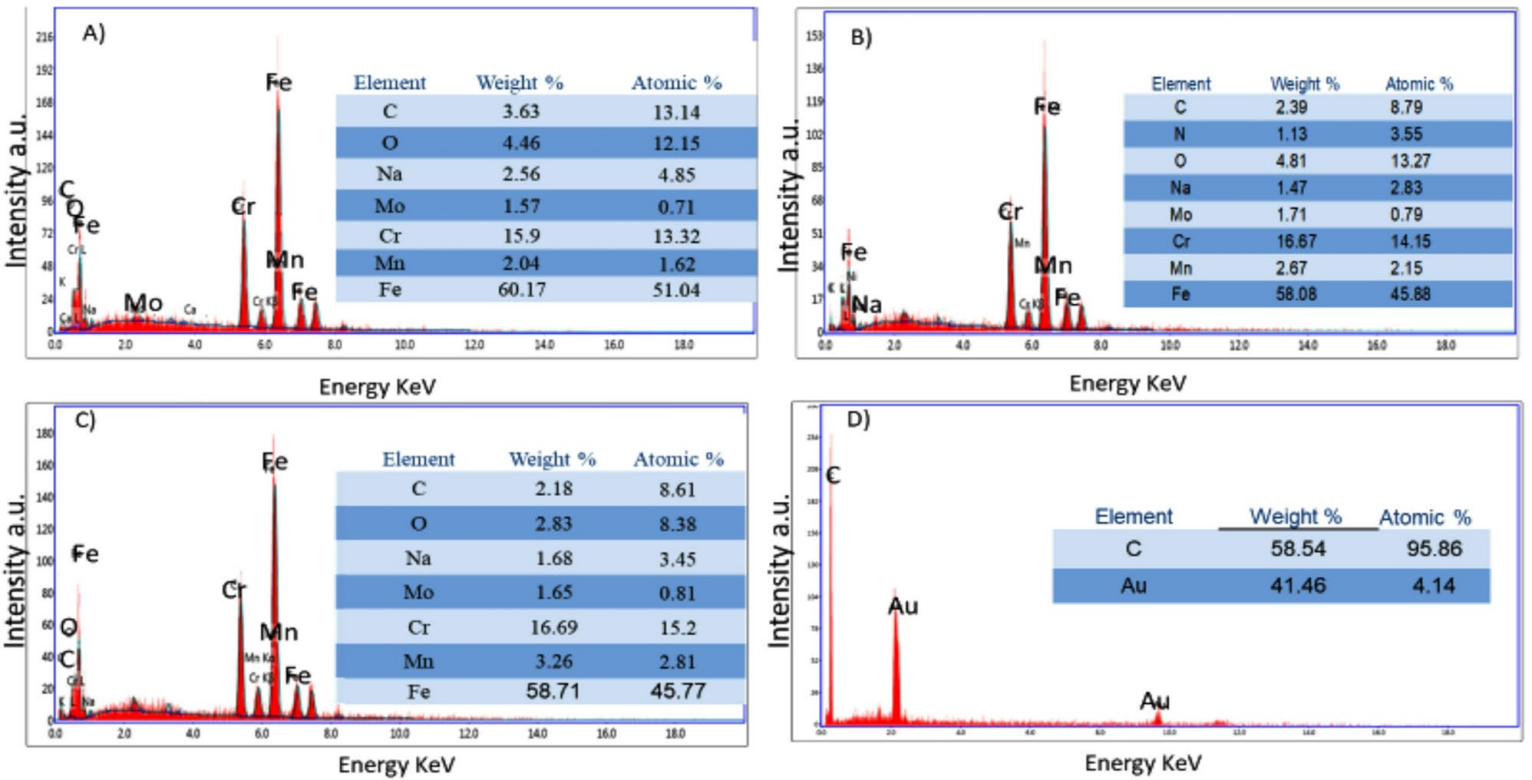
EDAX charts for the surface of the SS316L alloy in different media (**A**-**C**). SUS only (**A**), SUS containing *C. albicans* (**B**), SUS with *C. albicans* and Fluconazole (**C**). (**D**) EDAX chart for the surface of NAu-CPE

Figures 2(A–C) and 3(A–C) present the surface morphologies of SS316L as observed by SEM and analyzed by EDAX, respectively [28], under three different conditions: (A) in SUS as a control (blank), (B) in SUS containing *C. albicans*, and (C) in SUS with *C. albicans* and Fluconazole. In Fig. 2A, the alloy surface appears smooth and uniform with minimal pitting or surface defects, indicating strong corrosion resistance in the absence of microbial contamination. In contrast, Fig. 2B revealed scattered clusters of *C. albicans* adherent to the surface, suggesting biofilm formation and the onset of surface degradation.

Fig. 2 C illustrates the removal of biofilm from the surface following the introduction of Fluconazole, indicating its effectiveness as an antifungal agent. In Fig. 2D, the electrodeposited gold nanoparticles (NAu) appear as coral-like clusters distributed uniformly across the carbon paste (CP) substrate, a morphology that contributes to enhanced electrochemical performance. This observation is further supported by the EDAX spectrum shown in Fig. 3D. The EDAX analysis confirms that the elemental composition of the alloy closely matches the values previously reported in the experimental section and aligns with the expected structural features observed in the SEM images. Additionally, the EDAX spectra reveal the anticipated atomic percentages for each film analyzed.

**Fig. 4:**
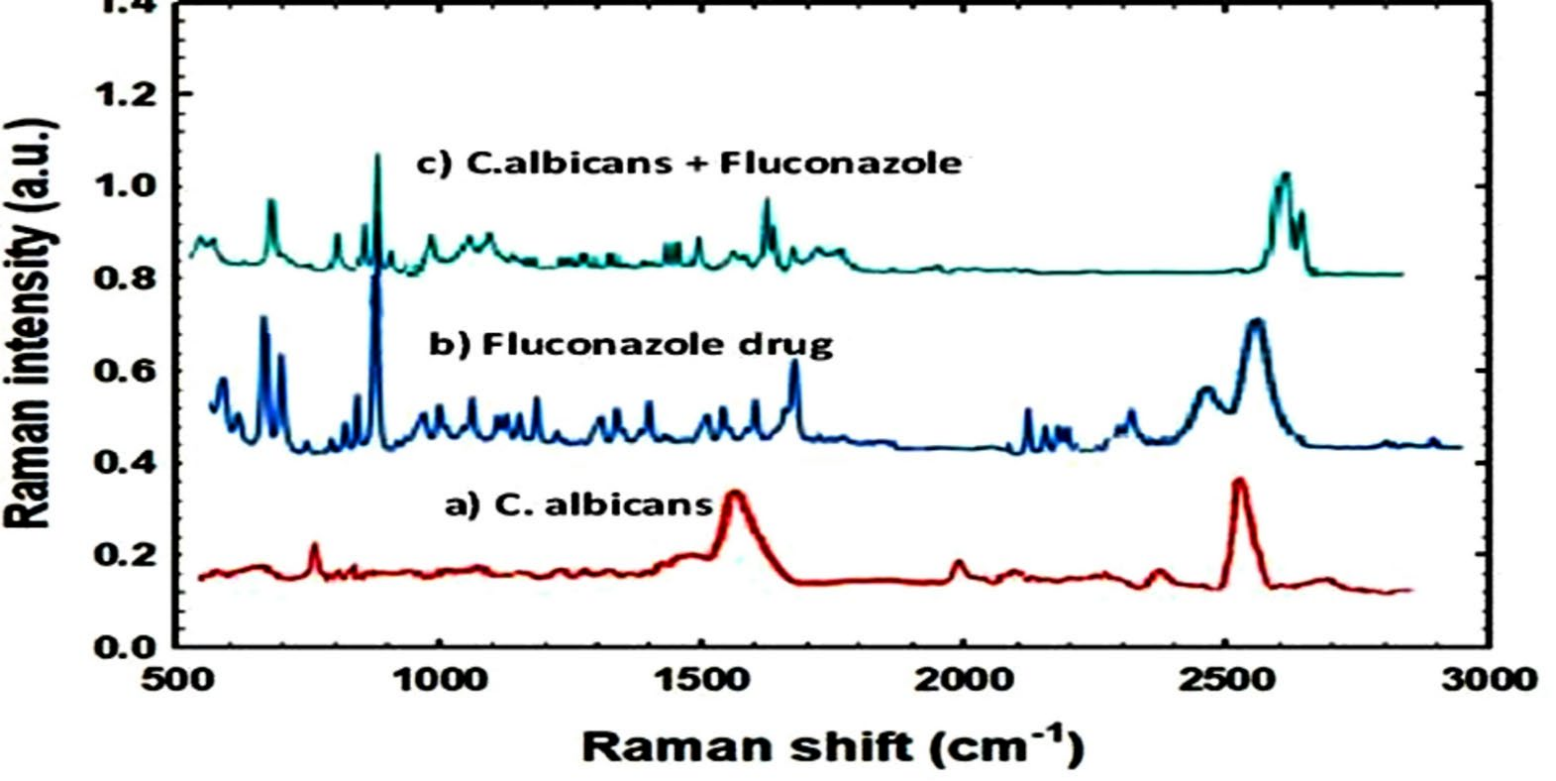
Raman spectrum for *C. albicans*, Fluconazole drug and *C. albicans* treated with Fluconazole drug

The interaction between *C. albicans* and the antifungal drug Fluconazole was studied using Raman spectroscopy to align with the paper’s primary objective of characterizing biochemical changes and evaluating drug effects at the molecular level. Raman spectroscopy provides a non-invasive method for identifying *C. albicans* through its unique molecular fingerprints, with specific spectral peaks serving as biochemical markers, as shown in Fig. 4. Fluconazole was spectrally characterized to identify its key vibrational features, such as principal group vibrations related to the triazole ring, which are consistent with its chemical structure. Upon treatment of *C. albicans* with Fluconazole, distinct spectral changes were observed. These included the appearance of new peaks, shifts in peak positions, and intensity changes indicating structural and biochemical alterations within the fungal cells. For instance, the oxidative degradation product of Fluconazole showed a peak at 1620 cm⁻¹, corresponding to C = N stretching, confirming the integrity of the triazole ring.

Furthermore, Fluconazole-induced stress affects the fungal cell wall, resulting in alterations in the polysaccharide region (1100–1200 cm⁻¹), as well as amide I (~ 1650 cm⁻¹) and amide II (~ 1550 cm⁻¹) bands, suggesting impacts on protein structure or folding. Lipid-related bands near 1440 cm⁻¹ (CH₂ bending) and 2850–2930 cm⁻¹ (CH stretching) also demonstrated changes, indicating membrane or lipid metabolism disruption. These spectral modifications confirm that Raman spectroscopy is an effective tool for monitoring the biochemical response of *C. albicans* to Fluconazole, thereby directly supporting the study’s objective of evaluating antifungal interactions using label-free, real-time vibrational analysis.

### EIS measurements

**Fig. 5:**
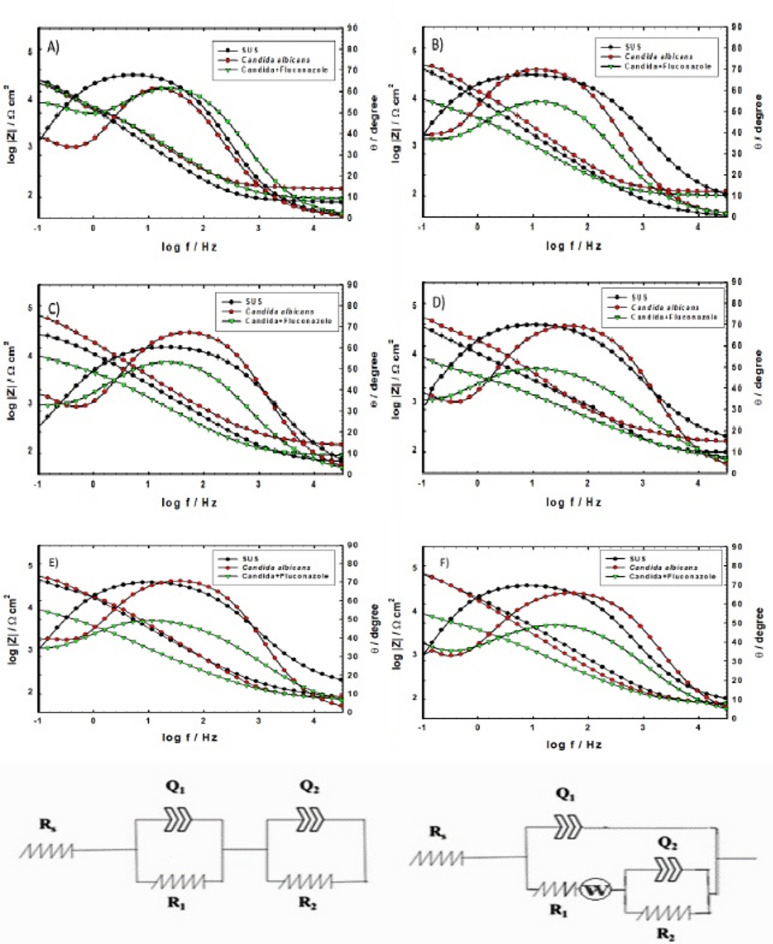
Bode plots of SS316L alloy in SUS without/with *C. albicans* and with the addition of Fluconazole after immersion for (**A**) 1 h, (**B**) 24 h, (**C**) 120 h, (**D**) 168 h, (**E**) 336 h, and (**F**) 408 h. Equivalent circuit models used for fitting

**Fig. 6:**
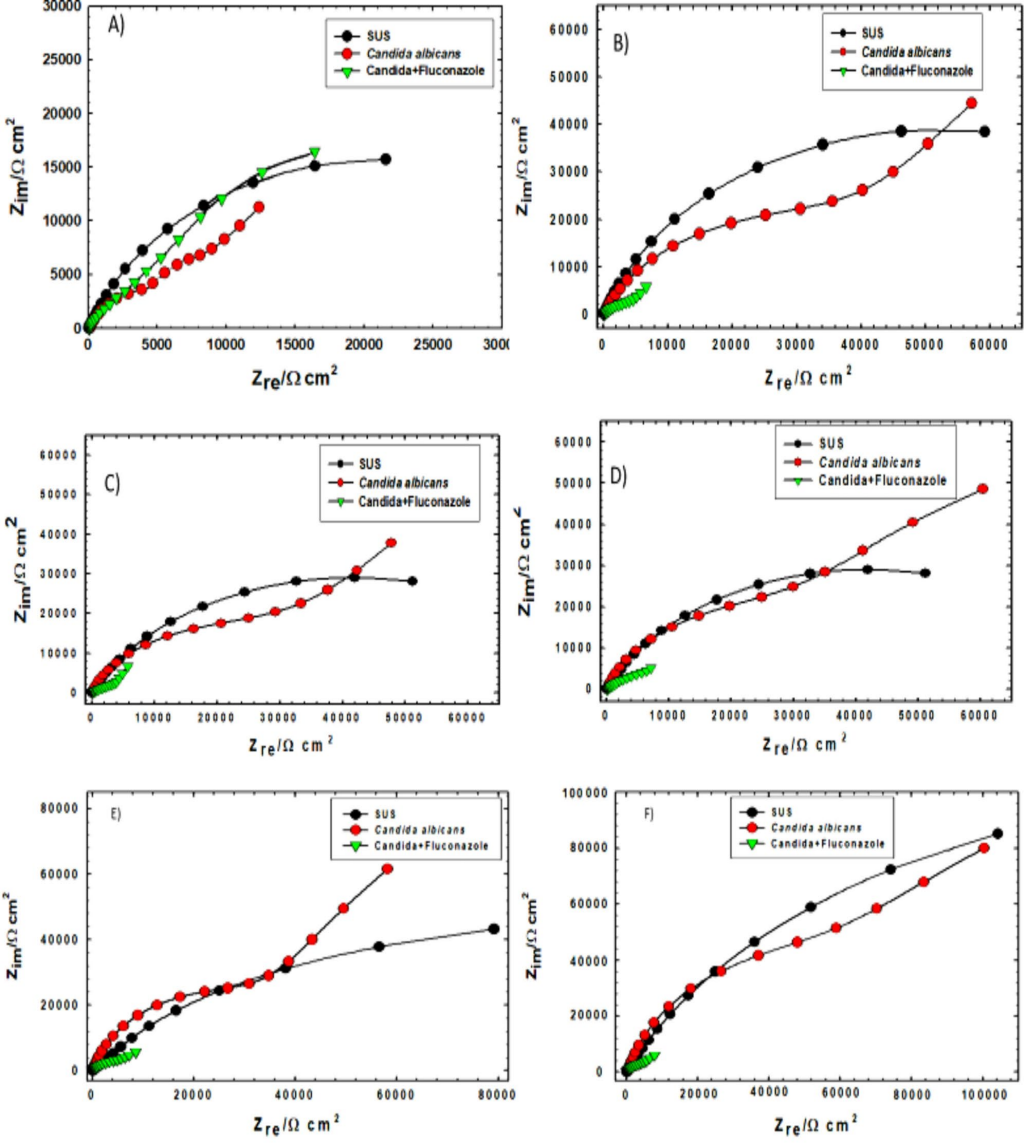
Nyquist plots of SS316L alloy in SUS without/with C. albicans and with the addition of Fluconazole after immersion for (**A**) 1 h, (**B**) 24 h, (**C**) 120 h, (**D**) 168 h, (**E**) 336 h, and (**F**) 408 h

Bode and Nyquist plots of the EIS spectra [29, 30] are shown in Figs. 5 and 6, respectively, for the SS316L in SUS with or without adding *C. albicans* and/or *C. albicans* with Fluconazole drug at 37 ⁰C for different immersion times of 1 h, 24 h, 120 h, 168 h, 336 h, and 408 h.

The structure of the SS316L provides a perfect surface for *C. albicans* adhesion, leading to the development of a biofilm owing to a complex interaction mechanism that may take place. *Candida*’s inhibitory impact increased as immersion time increased, perhaps delaying the pace at which SS316L alloy corrodes. The corrosion delay from *C. albicans* may be due to its acetaldehyde byproduct [31]; the electron donor species (which has an unshared lone pair of electrons on the oxygen heteroatom), and metabolites detected in the urine at the excretion mechanism may chelate well with the electrode surface, leading to corrosion rate decrease. After taking a suitable dose of Fluconazole, a glucuronidated form with the hydroxyl moiety and Fluconazole N-oxide byproducts from the drug exist from the interaction with *C. albicans* by chelation and forming a complex. Even the biofilm formed on the metal surface from the *C. albicans* is unstable and easily broken due to drug existence with a porous structure and starts further corrosion on the metal surface. At another scale, *C. albicans* has metabolites in this condition, which trigger the production of reactive oxygen species (ROS) [32, 33] from the mitochondria. This ROS is then converted to hydrogen peroxide, which then accumulates in the solution after cell wall lysis, which then induces corrosion to the alloy’s surface.

In Fig. 5, the black line represents the baseline electrochemical response of the SS316L alloy when immersed in SUS without any influence from *C. albicans* or Fluconazole. It shows moderate impedance, reflecting the inherent properties and passive film development of SS316L in a sterile synthetic urine environment. This line serves as a critical reference to understand how the presence of *C. albicans* and Fluconazole affects the alloy’s electrochemical stability. As seen from the phase diagram, the phase angle is stable and diffusion phenomena increased with time.

The red line represents the electrochemical behavior of SS316L in SUS in the presence of a *C. albicans* biofilm, without any antifungal treatment:

Stages from (1–24 h): observed a drop in impedance, indicating the initial stages of biofilm formation and a potential disruption or interaction with the alloy’s passive layer.

Stages from (120–408 h): As the biofilm matures, it shows an increase in both impedance magnitude (|Z|) and the phase angle with broadening of the phase angle diagram. This suggests that the developed biofilm acts as a physical diffusion barrier, hindering ion transport and thereby enhancing the alloy’s corrosion resistance over time. The *Candida* biofilm transformed from a potential initial corrosive influence to a protective one.

The green line illustrates the SS316L alloy’s response when simultaneously exposed to *C. albicans* biofilm and the antifungal drug Fluconazole:

Stages from (1–24 h): The impedance might be similar to or slightly lower than the red line, as Fluconazole begins to interfere with early fungal adhesion or biofilm formation.

Stages from (120–408 h): Generally, this line is expected to show lower impedance compared to the red line (without Fluconazole). This indicates that Fluconazole is actively destabilizing the *C. albicans* biofilm, lowering its protective barrier properties. As the biofilm breaks down, the alloy’s surface becomes more exposed to corrosive ions from the SUS, leading to reduced impedance and potentially enhanced material degradation. This highlights a complex trade-off between eliminating the fungal infection and maintaining the long-term stability of the metallic implant. As seen from the phase diagram, the phase angle and diffusion phenomena decreased.

Nyquist plots in Fig. 6 comprehensively illustrate the electrochemical impedance behavior of SS316L alloy in SUS, evolving across varied biological and pharmaceutical conditions over extended immersion times.

The black curves, representing the control (SS316L in SUS alone), consistently display the largest semicircular diameters at all time points. This signifies the highest polarization resistance and robust corrosion inhibition, attributed to the inherent stability of the passive oxide film on the alloy’s surface.

Conversely, the red curves, indicating the presence of *C. albicans*, initially show a decrease in impedance during the early stages (1–24 h), suggesting accelerated corrosion likely due to the initial disruption caused by biofilm formation. However, with prolonged exposure (120–408 h), these curves exhibit progressively increasing arc diameters. This suggests that the maturing biofilm functions as an effective diffusion barrier, impeding ionic exchange and providing a partial shield to the alloy’s surface against corrosive species in the SUS especially due to chelation.

In stark contrast, the green curves, depicting the combined effect of *C. albicans* and Fluconazole, consistently yield the lowest impedance values across all time intervals. This sustained reduction in impedance highlights that while Fluconazole effectively targets the fungal biofilm, it concurrently destabilizes its protective matrix.

Collectively, these findings underscore the complex role of biofilms in modulating corrosion behavior and emphasize the critical need for developing balanced antifungal strategies that do not compromise the long-term integrity of medical implants.

The best-fitting models for the done tests are shown in Fig. 5 with an average error of around 2%. It is a two-time constant model. Rs is the solution resistance, R1 is the outmost layer and R2 is the innermost layer. A constant-phase element (CPE) was introduced to avoid capacitance non-ideality and surface heterogeneity. Q1 is the CPE of the outermost layer and Q2 is for the innermost one.

The impedance of a CPE is defined by the expression Z_CPE_=1/Q(jω)^α^, where − 1 ≤ α ≤ 1. Here, Q represents the magnitude of the CPE, α is an empirical exponent that reflects surface non-uniformity, and ω is the angular frequency. W denotes the Warburg impedance, which arises due to diffusion processes. The fitted impedance data are presented in Table 2 [34].

Hence, it could be concluded that the alloy exposed to the SUS was in a passivated state. The solution resistance R_s_ was somewhat low owing to the presence of numerous salts in the developed medium. The dignified surface’s resistance proposes that the impedance is amplified because of the biofilm formation by the complex reaction of the fungi with the alloy’s surface. The increase in R₁ and R₂ values over time, especially for *C. albicans*, suggests that the biofilm formed acts as a physical barrier, forming a complex by chelation through electron pairs, limiting ion transport and enhancing corrosion resistance.

One can deduce that the insignificant discrepancy in Table 2 may result from the uneven biofilm formation, variability in microbial activity, or partial detachment of the biofilm during immersion, affecting impedance readings.

**Table 2:** The impedance parameters of immersed bare SS316L alloy in SUS with additives at 37 °C

SUS								
Time /h	Rs /Ω	Q1 /µF.cm-2	α 1	R1 /kΩ	Q2 /µF.cm-2	α 2	R2 /kΩ	W/kΩS^-1/2^
1	69.0	1.65	0.97	0.04	6.93	0.77	79.10	-
24	30.87	6.78	0.88	0.05	11.83	0.75	120.58	-
120	56.55	8.67	0.83	0.05	17.92	0.63	125.89	-
168	67.17	9.97	0.79	0.08	26.50	0.69	158.49	-
336	65.58	10.4	0.97	0.08	27.85	0.71	194.98	-
408	70.92	15.1	0.99	0.13	29.60	0.65	220.90	-
**SUS +** ***C. albicans***								
**Time /h**	**Rs /Ω**	**Q1 /µF.cm-2**	**α1**	**R1 /kΩ**	**Q2 /µF.cm-2**	**α 2**	**R2 /kΩ**	**W/kΩS** ^**-1/2**^
1	131.06	10.21	0.99	4.0	61.44	0.74	75.70	9.71
24	86.290	20.45	0.92	6.73	52.72	0.74	86.28	13.53
120	122.60	10.29	0.89	4.62	60.47	0.94	199.80	13.98
168	140.08	15.55	0.98	5.96	59.54	0.92	251.10	14.94
336	73.600	11.78	0.96	7.68	58.95	0.94	315.0	15.01
408	60.560	8.620	0.93	8.53	57.21	0.78	316.0	16.05
**SUS +** ***C. albicans*** **+ Fluconazole**								
**Time /h**	**Rs /Ω**	**Q1 /µF.cm-2**	**α 1**	**R1 /kΩ**	**Q2 /µF.cm-2**	**α 2**	**R2 /kΩ**	**W/kΩS** ^**-1/2**^
1	81.66	43.85	0.79	0.92	48.48	0.79	93.52	13.92
24	88.90	62.21	0.71	2.89	63.30	0.74	31.38	4.93
120	76.41	61.25	0.69	1.71	20.21	0.71	25.86	4.88
168	60.34	65.68	0.63	2.80	19.20	0.73	25.87	4.56
336	63.53	72.62	0.61	2.41	17.05	0.69	25.97	4.06
408	68.99	72.15	0.64	2.64	16.12	0.79	25.97	3.89

Also, EIS parameters showed an increase in R_1_ and R_2_ values with time for all and the best is for *C. albicans*.

### Potentiodynamic measurements

Tafel lines of the SS316L alloy [35] in SUS [36] with or without *C. albicans* and the addition of Fluconazole drug were developed after 168 h of immersion at 37 ⁰C, through a potential window of -0.6 to -0.2 V, and with a scan rate of 1 mV.s^-1^.

**Fig. 7:**
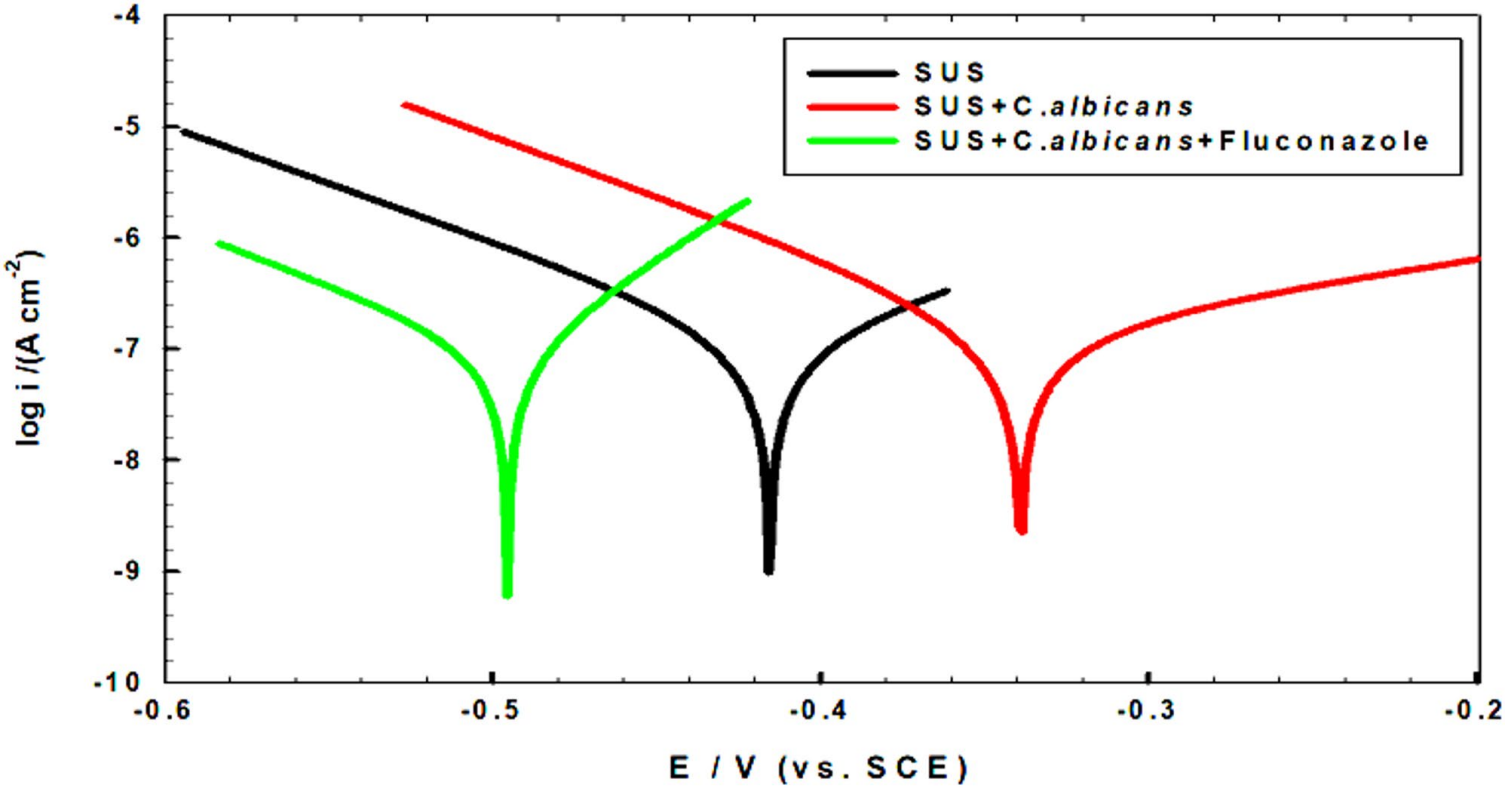
Tafel plots for SS316L alloy after immersion for 168 h in SUS with/without *C. albicans* and with the addition of Fluconazole at 37 °C

Figure 7 illustrates the Tafel lines, wherein the polarization parameters, including the corrosion potential (Ecorr) and corrosion current density (Icorr) acquired by extrapolation of the curves, are shown in Table 3 [37]. The results indicate that a more positive potential value (most passive film) was achieved from the existence of *C. albicans* > SUS > *C. albicans* + Fluconazole drug, and the anodic current in the presence of *C. albicans* is the smallest current. This indicates that the corrosion rate of the immersed SS316L alloy was inhibited in the presence of *C. albicans*. That may be due to the formation of microbial film on the surface of the alloy, which was then prevented due to the interaction with Fluconazole in the solution and these results make a good agreement with the results obtained from EIS. Also, as seen, the lowest cathodic branch, meaning the lowest hydrogen evolution, is for *C. albicans* with the lowest corrosion current density.

Considering the polarization resistance of the samples (R_p_ values) given in Table 3 by Eq. (1) (Stern-Geary equation) [38].

1 $$R_p=\frac{\beta a \beta c}{2.3 \operatorname{icorr}(\beta a+\beta c)}$$

Where i_corr_ is the corrosion current, and β_a_ and β_c_ are the anodic and cathodic Tafel slopes, correspondingly.

**Table 3:** Corrosion parameters of immersed SS316L alloy in SUS with/without additions for 168 h at 37 ⁰C

Medium	Ecorr /mV	icorr /µA cm-2	βa /mV.dec-1	βc /mV.dec-1	Rp /kΩcm2
SUS	-415.80	0.07	76.7	72.0	241.0
SUS + *C. albicans*	-339.16	0.06	79.9	53.2	247.95
SUS + *C. albicans +* Fluconazole	-495.58	0.08	50.0	80.2	176.19

It is so clear from Table 3 that the highest R_p_ is for SUS + *C. albicans* and this means the highest passivation.

### pH measurement

**Fig. 8:**
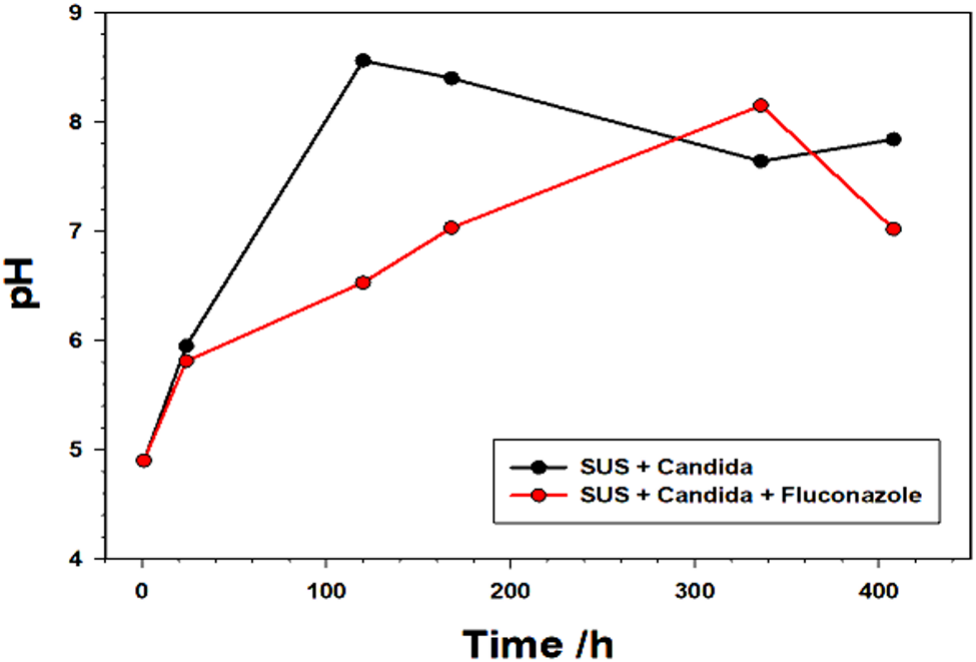
pH values of the solution medium in the presence of *C. albicans* growth and after treatment with the Fluconazole drug

Figure 8 displays the pH test results of the solution medium in the presence of *C. albicans* growth [39] and after treatment with the Fluconazole drug. It can be found that pH increases after the growth of *C. albicans* in SUS, with a value of about 8.6 and being alkaline, mainly for the reason of small amount of urea in SUS can also be decomposed by reaction of *C. albicans* to form ammonia, which dissolves in water to elevate the pH [40]. The strongest effect of *C. albicans* is at 168 h, which indicates that *C. albicans* has the strongest vitality on stainless steel immersed in SUS and the most vigorous metabolism at this time.

The addition of Fluconazole drug to the solution with *C. albicans* suppresses *C. albicans* growth and so the cell lysis products have the maximum elevation in pH after 408 h. Thus, in comparison to the case where the drug is not present, the increase in pH levels is minimal (Table 4).

### NAu-CPE sensor

**Fig. 9:**
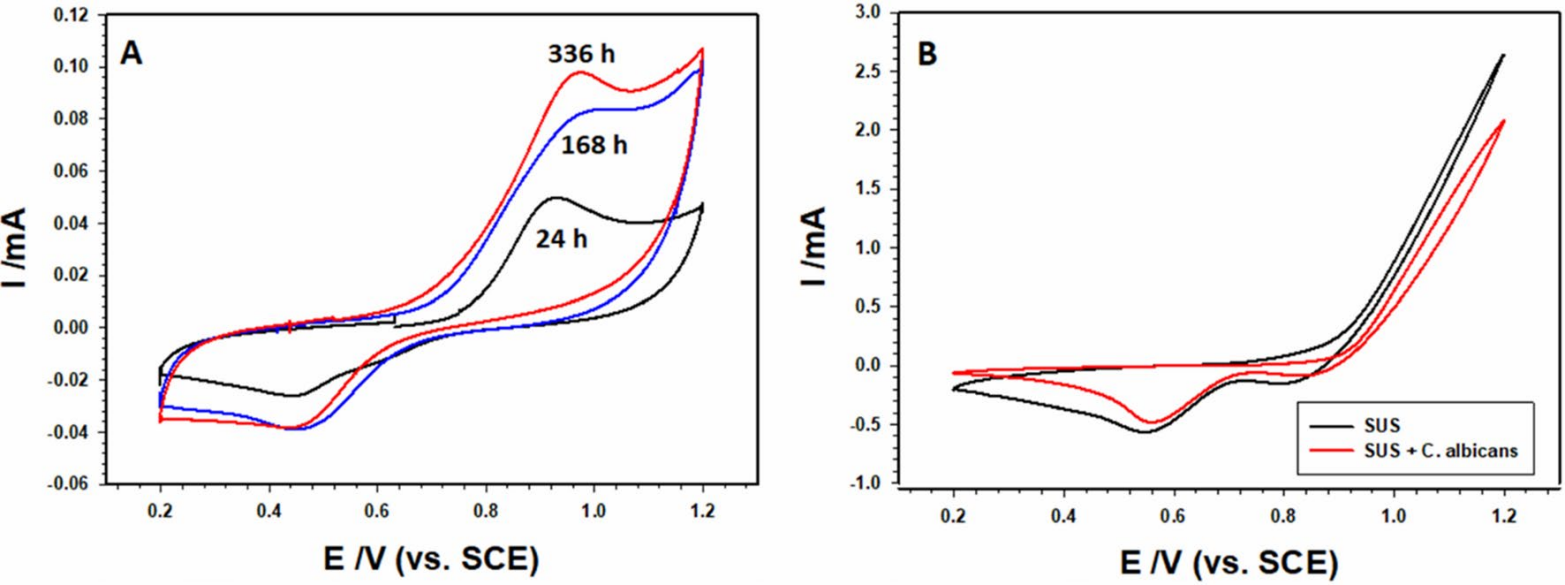
CVs for Fluconazole response by the NAu-CP sensor in the presence of *C. albicans* (**A**), The sensor’s response in SUS and SUS containing *C. albicans* (**B**)

Modification of CP with NAu particles enhanced the estimation of the Fluconazole drug in two different media, SUS and SUS containing *C. albicans*. CVs best explain this in Fig. 9A, in which a response peak at nearly 0.9 V is an indication of the Fluconazole [41]. Figure 9B represents the response of NAu-CPE in SUS and SUS containing *C. albicans*, respectively, this is to ensure that the response is for the drug only without any interference. Figure 9A shows an increase in the electrode’s response towards Fluconazole with time (24 h, 168 h, and 336 h), this can be accounted for the mechanism of action where at first the drug is attacking the *C. albicans* but as time proceeds the process decelerates and the drug becomes in the free form in the solution [42–45] where it becomes procurable to be detected by the NAu-CPE.

**Table 4:** Summary of measurement results and key findings

Measurement Type		Condition		Key Results / Findings	
SEM Morphology		SUS only		Smooth surface with few holes, indicating high corrosion resistance	
		SUS + *C. albicans*		Dispersed patches of *C. albicans* adhered to the surface.	
		SUS + *C. albicans* + Fluconazole		Improved film formation due to Fluconazole presence	
EDAX Elemental Analysis		SUS + additives		Elemental composition consistent with alloy: Fe, Cr, C, O, N, Mo, Mn, Na	
Raman Spectroscopy		*C. albicans*		Specific molecular signatures confirming fungal cell presence	
		Fluconazole		Characteristic vibrational peaks matching the drug structure	
		*C. albicans* + Fluconazole		Peak shifts and new peaks indicating biochemical changes and oxidative degradation	
EIS (Electrochemical Impedance Spectroscopy)		SUS ± *C. albicans* ± Fluconazole		Increased impedance (R₁, R₂) with *C. albicans* showing biofilm formation and corrosion resistance; Fluconazole reduces biofilm stability	
Tafel Polarization		SUS ± *C. albicans* ± Fluconazole		Highest corrosion resistance (Rp) in SUS + *C.* *albicans*; Fluconazole reduces corrosion resistance	
pH Measurement		SUS + *C. albicans*		pH rises to ~ 8.6 due to ammonia from urea decomposition	
		SUS + *C. albicans* + Fluconazole		Lower pH increases due to fungal growth suppression	
NAu-CPE Sensor (CV Analysis)		SUS + *C. albicans* +Fluconazole		Fluconazole peak ~ 0.9 V; signal increases over time as the drug is released from fungal cells	

## Conclusions

- ○ In this study, the formation of microbial films of *C. albicans* in the presence and absence of Fluconazole on the surface of SS316L alloy implant as ureteric stent was investigated with scanning electron microscopy and electrochemical analysis techniques.
- ○ The in-vitro immersion experiments and electrochemical corrosion measurements indicated that the combined action of metabolites produced by *C. albicans* without adding a drug passivates the metal more than when interacting with the drug and simulated urine.
- ○ The formation of microbial film can lead to changes in the corrosion electrochemistry of SS316L alloy.
- ○ A more positive potential value was achieved from the existence of *C. albicans* > SUS >. *C. albicans* Fluconazole drug, and the anodic current in the presence of *C. albicans* is the smallest.
- ○ EIS parameters showed an increase in R1 and R2 values with time for all and the best is for *C. albicans*.
- ○ Completely different surface product coverage layer morphologies were represented by Raman spectroscopy, SEM and EDAX.
- ○ Our findings directly address the critical gap in understanding the electrochemical behavior of SS316L stents in *Candida*-infected urinary environments, providing novel insights into biofilm-material interactions.

## Data Availability

Data is provided within the manuscript or supplementary information files.

## References

[1] Shoshany O, Erlich T, Golan S, Kleinmann N, Baniel J, Rosenzweig B, Eisner A, Mor Y, Ramon J, Winkler H, Lifshitz D. Ureteric stent versus percutaneous nephrostomy for acute ureteral obstruction - clinical outcome and quality of life: a bi- center prospective study. BMC Urol. 2019;19:1–7. 10.1186/s12894-019-0510-4. 31455309 10.1186/s12894-019-0510-4 PMC6712738

[2] Gao X, Di X, Chen G, Wang W, Peng L, Chen J, Wei X. Metal ureteral stents for ureteral stricture: 2 years of experience with 246 cases. Int J Surg. 2024;110:66–71. 10.1097/JS9.0000000000000841. 37812177 10.1097/JS9.0000000000000841 PMC10793778

[3] Kulkarni R. Metallic stents in the management of ureteric strictures. Indian J Urol. 2014;30:65–72. 10.4103/0970-1591.124210. 24497686 10.4103/0970-1591.124210 PMC3897057

[4] Al-Aown A, Kyriazis I, Kallidonis P, Kraniotis P, Rigopoulos C, Karnabatidis D, Petsas T. Liatsikos, ureteral stents: new ideas, new designs, ther. Adv Urol. 2010;2:85–92. 10.1177/1756287210370699. 10.1177/1756287210370699 PMC3126070 21789086

[5] Fekry AM, El-Kamel RS, Ghoneim AA. Electrochemical behavior of surgical 316L stainless steel eye glaucoma shunt (Ex-PRESS) in artificial aqueous humor. J Mater Chem B. 2016;4:4542–8. 10.1039/C6TB00712K. 32263397 10.1039/c6tb00712k

[6] Bruschi S, Pezzato L, Ghiotti A, Dabalà M, Bertolini R. Effectiveness of using low- temperature coolants in machining to enhance durability of AISI 316L stainless steel for reusable biomedical devices. J Manuf Processes. 2019;39:295–304. 10.1016/j.jmapro.2019.02.003.

[7] Hermawan H, Ramdan D, Djuansjah JRP. Metals for biomedical applications. In: Rezai RF, editor. Biomedical engineering-from theory to applications. Croatia: InTech; 2011. pp. 411–30. 10.5772/19033.

[8] Flores-Mireles AL, Walker JN, Caparon M, Hultgren SJ. Urinary tract infections: epidemiology, mechanisms of infection and treatment options. Nat Rev Microbiol. 2015;13:269–84. 10.1038/nrmicro3432. 25853778 10.1038/nrmicro3432 PMC4457377

[9] Fisher JF, Kavanagh K, Sobel JD, Kauffman CA, Newman CA. Candida urinary tract infection: pathogenesis. Clin Infect Dis. 2011;suppl6:S437–51. 10.1093/cid/cir110. 10.1093/cid/cir110 21498837

[10] Werneburg GT, Hettel D, Lundy SD, Adler A, De S, Mukherjee SD, Rackley RR, Shoskes DA, Miller AW. Ureteral stents harbor complex biofilms with rich Microbiome-Metabolite interactions. J Urol. 2023;209:950–62. 10.1097/JU.0000000000003200. 36724057 10.1097/JU.0000000000003200

[11] Mainali P, Luitel P, Paudel S, Thapaliya I, Sharma UK, Chapagain S, Gautam P, Luitel BR, Chalise PR. Risk factors for bacterial stent colonization in patients with a double J ureteral stent: a prospective study. Annals Med Surg. 2024;86:7023–8. 10.1097/MS9.0000000000002683. 10.1097/MS9.0000000000002683 PMC11623820 39649891

[12] Zhang JM, Liu J, Wang K, Zhang X, Zhao T, Luo HM. Observations of bacterial biofilm on ureteral stent and studies on the distribution of pathogenic Bacteria and drug resistance. Urol Int. 2018;101:320–6. 10.1159/000490621. 30212821 10.1159/000490621

[13] Pfaller MA, Diekema DJ, Gibbs DL, Newell VA, Ellis D, Tullio V, Rodloff A, Fu W, Ling TA. Results from the ARTEMIS DISK global antifungal surveillance study, 1997 to 2007: a 10.5-year analysis of susceptibilities of Candida species to fluconazole and voriconazole as determined by CLSI standardized disk diffusion. J Clin Microbiol. 2010;4:1366–77. 10.1128/jcm.02117-09. 10.1128/JCM.02117-09 PMC2849609 20164282

[14] Berkow EL, Lockhart SR. Fluconazole resistance in Candida species: a current perspective. Infect Drug Resist. 2017;10:237–45. 10.2147/IDR.S118892. 28814889 10.2147/IDR.S118892 PMC5546770

[15] Antonin VS, Santos MC, Garcia-Segura S, Brillas E. Electrochemical incineration of the antibiotic Ciprofloxacin in sulfate medium and synthetic urine matrix. Water Res. 2015;83:31–41. 10.1016/j.watres.2015.05.066. 26117371 10.1016/j.watres.2015.05.066

[16] Atta NF, Fekry AM, Hassaneen HM. Corrosion inhibition, hydrogen evolution and antibacterial properties of newly synthesized organic inhibitors on 316L stainless steel alloy in acid medium. Int J Hydrogen Energy. 2011;36:6462–71. 10.1016/j.ijhydene.2011.02.134.

[17] Mohammad KA, Zainudin ES, Salit MS, Zahari NI, Ali A. Experimental determination of the fatigue behavior of austenitic 316L stainless steel under fatigue and Creep-Fatigue tests at high temperature. Int J Met Steel Res Technol. 2013;1:1–11.

[18] Monteiro RD, Wetering JVD, Krawczyk B, Engelberg DL. Corrosion behaviour of type 316L stainless steel in hot caustic aqueous environments. Met Mater Int. 2020;26:630–40. 10.1007/s12540-019-00403-2.

[19] Shet N, Nazareth R, Suchetan PA. Corrosion Inhibition of 316 stainless steel in 2 M HCl by 4-{[4-(dimethylamino)benzylidene]amino}-5-methyl-4H-1,2,4-triazole-3-thiol. Chem Data Collect. 2019;20:100209. 10.1016/j.cdc.2019.100209.

[20] Abdel Ghanyl NA, El-Shenawy AE, Hussien WAM. The inhibitive effect of some amino acids on the corrosion behaviour of 316L stainless steel in sulfuric acid solution. Mod Appl Sci. 2011;5:19. 10.5539/mas.v5n4p19.

[21] Cardona C, Torres AA, Miranda-Vidales JM, Pérez JT, González-Chávez MM, Herrera-Hernández H, Narváez L. Assessment of dimethylbenzodiimidazole as corrosion inhibitor of austenitic stainless steel grade 316L in acid medium. Int J Electrochem Sci. 2015;10:1966–78. 10.1016/S1452-3981(23)04821-6.

[22] Abdel Gawad S, Nasr A, Fekry AM, Filippov LO. Electrochemical and hydrogen evolution behaviour of a novel nano-cobalt/nano-chitosan composite coating on a surgical 316L stainless steel alloy as an implant. Int J Hydrogen Energy. 2021;46:18233–41. 10.1016/j.ijhydene.2021.03.018.

[23] El-Kamel RS, Fekry AM. Enhanced modified poly-tyrosine voltammetric sensor for the quantification detection of salivary Pepsin. Int J Biol Macromol. 2024;277:134178. 10.1016/j.ijbiomac.2024.134178. 39067726 10.1016/j.ijbiomac.2024.134178

[24] Mamdouh S, Shehata M, Fekry AM, Ameer MA. Electro-polymerization of modified carbon paste sensor for detecting Azithromycin. Sci Rep. 2025;15:980. 10.1038/s41598-024-79614-6. 39762252 10.1038/s41598-024-79614-6 PMC11704252

[25] Mamdouh S, Shehata M, Fekry AM, Ameer MA. Graphite-based sensor amended with fumed silica for electrodetection of Azithromycin. Can J Chem. 2022;8:589–600. 10.1139/cjc-2021-0295.

[26] Shehata M, Fekry AM, Walcarius A. Moxifloxacin hydrochloride electrochemical detection at gold nanoparticles modified Screen-Printed electrode. Sensors. 2020;20:2797. 10.3390/s20102797. 32423013 10.3390/s20102797 PMC7287685

[27] Hamed SM, Zedan AF, Abdelhady HH, Mohamed NM, Fekry AM. Graphitic carbon nitride/titania nanotube arrays for photoelectrochemical oxidation of methanol under visible light. Int J Hydrogen Energy. 2024;90:918–30. 10.1016/j.ijhydene.2024.09.292.

[28] Akram W, Rafique AF, Maqsood N, Khan A, Badshah S, Khan RU. Characterization of PTFE film on 316L stainless steel deposited through spin coating and its anticorrosion performance in multi acidic mediums. Materials. 2020;13:388. 10.3390/ma13020388. 31947700 10.3390/ma13020388 PMC7014069

[29] Zatkalíková V, Halanda J, Vaňa D, Uhríčik M, Markovičová L, Štrbák M, Kuchariková L. Corrosion resistance of AISI 316L stainless steel biomaterial after plasma immersion ion implantation of nitrogen. Materials. 2021;14:6790. 10.3390/ma14226790. 34832193 10.3390/ma14226790 PMC8620768

[30] Nicho K, Yokoyama K. Marked degradation of tensile properties induced by plastic deformation after interactions between Strain-Induced martensite transformation and hydrogen for type 316L stainless steel, metals. 10 (2020) 928. 10.3390/met10070928

[31] Zeng F, Li Y, Chen K, Li G, Liu C, Wang L, Li L, Qu Q. Adsorption of Candida albicans on Ti-6Al-4V surface and its corrosion effects in artificial saliva. Bioelectrochemistry. 2022;148:108248. 10.1016/j.bioelechem.2022.108248. 35988504 10.1016/j.bioelechem.2022.108248

[32] Pradhan A, Avelar GM, Bain JM, Childers DS, Larcombe DE, Netea MG, Shekhova E, Munro CA, Brown GD, Erwig LP, Gow NA. Hypoxia promotes immune evasion by triggering β-glucan masking on the Candida albicans cell surface via mitochondrial and cAMP-protein kinase A signalling, MBio. 6 (2018) 10–1128. 10.1128/mbio.01318-18 10.1128/mBio.01318-18 PMC6222127 30401773

[33] Ruiz-Herrera J, Victoria Elorza M, Valentín E, Sentandreu R. Molecular organization of the cell wall of Candida albicans and its relation to pathogenicity. FEMS Yeast Res. 2006;1:14–29. 10.1111/j.1567-1364.2005.00017.x. 10.1111/j.1567-1364.2005.00017.x 16423067

[34] Elkamel RS, Fekry AM, Ghoneim AA, Filippov LO. Electrochemical corrosion behaviour of AZ91E magnesium alloy by means of various nanocoatings in aqueous peritoneal solution: in vitro and in vivo studies. J Mater Res Technol. 2022;17:828–39. 10.1016/j.jmrt.2022.01.007.

[35] Arwati IGA, Alfattah M, Maryani Y, Suprihatiningsih W, Yuliarty P. Analysis of the corrosion rate of SS 316l metal in a tsunami detection tool in seawater media. BIO Web Conf. 2025;159:07003. 10.1051/bioconf/202515907003.

[36] Nagalakshmi R, Prabasheela B. Surface characterization of SS 316L in synthetic urine in presence of bile salt. Int J Res Appl Sci Eng Technol. 2018;6:2702–9. 10.22214/ijraset.2018.5442.

[37] Heakal FE, Fekry AM, Jibril MA. Electrochemical behaviour of the Mg alloy AZ91D in Borate solutions. Corros Sci. 2011;4:1174–85. 10.1016/j.corsci.2010.11.040.

[38] El-Kamel RS, Ghoneim AA, Fekry AM. Electrochemical, biodegradation and cytotoxicity of graphene oxide nanoparticles/polythreonine as a novel nano-coating on AZ91E Mg alloy staple in gastrectomy surgery. Mater Sci Eng C. 2019;103:109780. 10.1016/j.msec.2019.109780. 10.1016/j.msec.2019.109780 31349436

[39] Zheng LIU, ZHANG LM, REN DC, MA AL, JI HB, ZHENG YG. Corrosion behavior of Ti– 6Al– 4V alloy fabricated by selective laser melting in simulated spent fuel reprocessing environment. Trans Nonferrous Met Soc China. 2024;34:2167–80. 10.1016/S1003-6326(24)66532-5.

[40] Vylkova S, Carman AJ, Danhof HA, Collette JR, Zhou H, Lorenz MC. The fungal pathogen Candida albicans autoinduces hyphal morphogenesis by Raising extracellular pH, mBio. 2 (2011) e00055–11. 10.1128/mbio.00055-11 10.1128/mBio.00055-11 PMC3101780 21586647

[41] Zad ZR, Davarani SS, Taheri A, Bide Y. A yolk shell Fe3O4@ PA-Ni@ pd/chitosan nanocomposite-modified carbon ionic liquid electrode as a new sensor for the sensitive determination of fluconazole in pharmaceutical preparations and biological fluids. J Mol Liq. 2018;253:233–40. 10.1016/j.molliq.2018.01.019.

[42] Salama NN, Azab SM, Mohamed MA, Fekry AM. A novel methionine/palladium nanoparticle modified carbon paste electrode for simultaneous determination of three antiparkinson drugs. RSC Adv. 2015;5:14187–95. 10.1039/C4RA15909H.

[43] Medany SS, Ahmad YH, Fekry AM. Experimental and theoretical studies for corrosion of molybdenum electrode using streptomycin drug in phosphoric acid medium. Sci Rep. 2023;13:4827. 10.1038/s41598-023-31886-0. 36964162 10.1038/s41598-023-31886-0 PMC10038993

[44] Hendawy HA, Eldin GM, Fekry AM. A metal substituted nano ferrite (M = Zn, cu, Fe and mn; x = 0 and 0.5)] improved Screen-Printed electrode for anodic determination of Toldimfos sodium. Microchem J. 2023;185:108267. 10.1016/j.microc.2022.108267.

[45] Fekry AM, Azab SM, Abou Attia FM, Ibrahim NS, Mohamed GG. An innovative sensor for the electrochemical determination of the new melatonergic antidepressant drug agomelatine. Measurement. 2021;186:110160. 10.1016/j.measurement.2021.110160.

